# CHARMS: A CNN-Transformer Hybrid with Attention Regularization for MRI Super-Resolution

**DOI:** 10.3390/s26020738

**Published:** 2026-01-22

**Authors:** Xia Li, Haicheng Sun, Tie-Qiang Li

**Affiliations:** 1 College of Information Engineering, China Jiliang University, Hangzhou 314423, China; 2 School of Medical Imaging, Fujian Medical University, Fuzhou 350005, China; 3 Department of Medical Radiation Physics and Nuclear Medicine, Karolinska University Hospital, 171 76 Stockholm, Sweden; 4 Department of Clinical Science, Intervention and Technology, Karolinska Institutet, 171 77 Stockholm, Sweden

**Keywords:** MRI super-resolution, lightweight network, CNN-Transformer hybrid, attention regularization, reverse residual attention, clinical deployment

## Abstract

**Highlights:**

**What are the main findings?**

- CHARMS, a lightweight CNN-Transformer hybrid (~1.9 M parameters, ~30 GFLOPs), outperforms state-of-the-art lightweight MRI super-resolution models (EDSR, PAN, W2AMSN-S, and FMEN) by 0.1–0.6 dB PSNR and up to 1% SSIM at ×2/×4 upscaling while reducing inference time by ~40%.
- With cross-field fine-tuning on only twenty subjects, CHARMS upgrades clinical 3T MRI to near-7T quality, yielding ~6 dB PSNR and 0.12 SSIM gains over native 3T scans across T1w/T2w contrasts.

**What are the implications of the main findings?**

- Near-real-time performance (~11 ms/slice enabling ~1.6–1.9 s processing per 3D brain volume on RTX 4090) and small model size enable practical deployment in clinical workstations, online reconstruction pipelines, and resource-constrained environments including low-field and portable MRI scanners.
- Superior fidelity–efficiency balance paves the way for shorter scan times, reduced motion artifacts, and 7T-like diagnostic quality from standard 3T systems without additional hardware.

**Abstract:**

Magnetic resonance imaging (MRI) super-resolution (SR) enables high-resolution reconstruction from low-resolution acquisitions, reducing scan time and easing hardware demands. However, most deep learning-based SR models are large and computationally heavy, limiting deployment in clinical workstations, real-time pipelines, and resource-restricted platforms such as low-field and portable MRI. We introduce CHARMS, a lightweight convolutional–Transformer hybrid with attention regularization optimized for MRI SR. CHARMS employs a Reverse Residual Attention Fusion backbone for hierarchical local feature extraction, Pixel–Channel and Enhanced Spatial Attention for fine-grained feature calibration, and a Multi-Depthwise Dilated Transformer Attention block for efficient long-range dependency modeling. Novel attention regularization suppresses redundant activations, stabilizes training, and enhances generalization across contrasts and field strengths. Across IXI, Human Connectome Project Young Adult, and paired 3T/7T datasets, CHARMS (~1.9M parameters; ~30 GFLOPs for 256 × 256) surpasses leading lightweight and hybrid baselines (EDSR, PAN, W2AMSN-S, and FMEN) by 0.1–0.6 dB PSNR and up to 1% SSIM at ×2/×4 upscaling, while reducing inference time ~40%. Cross-field fine-tuning yields 7T-like reconstructions from 3T inputs with ~6 dB PSNR and 0.12 SSIM gains over native 3T. With near-real-time performance (~11 ms/slice, ~1.6–1.9 s per 3D volume on RTX 4090), CHARMS offers a compelling fidelity–efficiency balance for clinical workflows, accelerated protocols, and portable MRI.

## 1. Introduction

Magnetic resonance imaging (MRI) is a cornerstone modality in clinical diagnostics and biomedical research, offering superior soft tissue contrast without ionizing radiation. However, acquiring high-resolution (HR) MRI often requires long scan durations or high-field systems, which increase costs, patient discomfort, and vulnerability to motion artifacts. Super-resolution (SR) reconstruction [1,2,3] seeks to mitigate these limitations by recovering HR images from low-resolution (LR) inputs, thereby enhancing spatial detail without extending acquisition time.

Deep learning has substantially advanced SR reconstruction by enabling end-to-end mappings between LR and HR domains. Early 2D convolutional neural network (CNN) models such as SRCNN [4], VDSR [5], EDSR [6], and RCAN [7] achieved strong gains in image fidelity through deep feature extraction and residual learning. These methods demonstrated the potential of CNNs to recover fine structural details but often depended on increasingly deep or wide architecture. As a result, they introduced high computational cost, large memory demand, and limited suitability for real-time or near-real-time MRI workflow.

Subsequent efforts introduced lightweight 2D CNN variants, including MobileNet [8], ShuffleNet [9], and ESPNet [10], and explored depthwise separable convolutions and channel decomposition to reduce parameters while preserving performance. Although effective for accelerating SR networks, these designs generally lacked mechanisms for modeling long-range spatial relationships, which are essential for reconstructing complex anatomical structures.

To address these limitations, we propose CHARMS (CNN-Transformer Hybrid with Attention Regularization for MRI Super-Resolution), a compact and efficient SR framework tailored for MRI. CHARMS integrates a Reverse Residual Attention Fusion (RRAF) backbone [11] for hierarchical feature extraction with Pixel–Channel Attention (PCA) [12] and Enhanced Spatial Attention (ESA) modules [13] for fine-grained feature calibration. Multi-Depthwise Dilated Transformer Attention (MDDTA) module [14] captures long-range dependencies with linear computational complexity, while an attention regularization mechanism reduces redundancy and stabilizes training (see Appendix A for a glossary of acronyms and brief descriptions of the main architectural modules).

Extensive experiments on multiple open-access MRI datasets, including IXI [15], Human Connectome Project Young Adult (HCP-YA) [16], and paired 3T-7T datasets [17,18], demonstrate that CHARMS achieves superior reconstruction accuracy with significantly fewer parameters and lower inference latency compared to existing CNN- and Transformer-based SR models. By balancing representational power with computational efficiency, CHARMS advances the integration of deep SR frameworks into modern MRI acquisition and reconstruction pipelines.

This study addresses the following research questions: **RQ1:** Can a lightweight CNN-Transformer hybrid outperform existing MRI SR models in fidelity–efficiency trade-off? **RQ2:** How does attention regularization improve training stability and cross-contrast/field-strength generalization? **RQ3:** What is the clinical potential of limited-data cross-field fine-tuning for upgrading standard 3T scans to near-7T quality?

## 2. Related Work

### 2.1. CNN-Based MRI Super-Resolution

Early deep SR models relied heavily on 2D CNNs. SRCNN pioneered end-to-end learning with a simple three-layer architecture, while VDSR and EDSR introduced residual learning and deeper networks for enhanced detail recovery. RCAN advanced this further by incorporating channel attention to adaptively recalibrate features. Lightweight variants, inspired by MobileNet, ShuffleNet, and ESPNet, employed depthwise separable convolutions and channel shuffling to reduce parameters, achieving faster inference suitable for clinical constraints. Despite these efficiency gains, pure CNN approaches often struggle with modeling long-range spatial dependencies critical for reconstructing extended anatomical structures in brain MRI.

### 2.2. Attention Mechanisms in SR

Attention modules have significantly boosted SR performance [19,20]. Channel [7] and spatial attention [19] modules improved intra-feature relevance by adaptively weighting informative regions, while residual attention blocks [7,12,19,20,21,22] helped capture hierarchical contextual cues. More recently, Transformer-based architectures [23] introduced self-attention to model long-range dependencies across the entire image plane [24,25,26,27]. These developments produced notable improvements in reconstruction accuracy and generalization. Representative hybrid CNN-Transformer SR models, such as SwinIR [28], Restormer [27], and TTVSR [29], demonstrated the value of combining convolutional locality with global context modeling. However, many of these hybrid approaches [30,31,32,33] rely on computationally expensive attention operations, redundant attention activations, or complex multi-stage pipelines, which hinder their scalability to high-resolution volumetric MRI and limit deployment in resource-constrained clinical environments.

### 2.3. Transformer and Hybrid Architectures

The introduction of Transformers revolutionized natural image SR through global self-attention, as exemplified by SwinIR (shifted-window attention), Restormer (multi-head channel attention), and TTVSR. These hybrid CNN-Transformer models combine inductive biases of convolutions with Transformer’s long-range modeling, yielding superior fidelity. In MRI SR, variants such as SuperFormer, REHRSeg, and physics-informed hybrids have emerged, often extending to multi-contrast or anisotropic data. Very recent advances include VMamba-Transformer hybrids (e.g., MRISR [34,35,36,37,38,39]) for low-field enhancement and diffusion-guided models that leverage generative sampling for texture-rich reconstruction. Although powerful, these methods frequently involve high parameter counts (>10 M), iterative sampling, or quadratic attention complexity, limiting real-time applicability.

Transformer-enhanced frameworks often incur prohibitive computational overhead [24,27,33,40]. A key challenge is how to design an efficient hybrid model that preserves local representational strength, captures long-range dependencies, and avoids redundant or unstable attention behaviors. Addressing this challenge is critical for advancing practical MRI SR, especially in hardware-limited settings where latency, memory footprint, and energy consumption are essential constraints. In addition to these CNN and Transformer-based methods, several very recent models have further extended MRI SR capabilities. MRISR [41] employs a VMamba-Transformer architecture to enhance texture reconstruction in low-field MRI, while a 2D slice-wise diffusion model [42] demonstrates the strong generative capacity of diffusion sampling for high-fidelity SR. Although effective, these approaches tend to be computationally demanding, reinforcing the need for lightweight and stable SR frameworks.

### 2.4. Diffusion Models and Emerging Trends

Diffusion-based SR frameworks have recently demonstrated exceptional perceptual quality by treating super-resolution as iterative denoising. Models exploiting latent diffusion or physics-informed guidance achieve impressive detail recovery, particularly in multi-parametric or low-SNR settings. However, their reliance on dozens to hundreds of sampling steps renders them computationally intensive, often unsuitable for clinical workflows demanding sub-second latency. Recent advances have extended MRI SR with diffusion models for high-fidelity generation [35,42,43,44,45,46] and Transformer hybrids for multi-contrast handling [44,47]. However, these often prioritize perceptual quality over computational efficiency, with high iteration counts or parameters (>10 M), limiting real-time clinical use [48,49,50]. CHARMS addresses this gap by integrating lightweight CNN-Transformer fusion with attention regularization, achieving SOTA efficiency–fidelity balance for portable and cross-field MRI [35,42,43,44,45,46,47,51].

CHARMS distinguishes itself by integrating lightweight convolutional processing with linear complexity-dilated Transformer attention and explicit regularization, achieving competitive or superior fidelity at a fraction of the computational cost of contemporary Transformer hybrids and diffusion models. This positions CHARMS as a practical solution for resource-constrained and cross-field MRI super-resolution.

## 3. Materials and Methods

### 3.1. CHARMS Framework

**Figure 1:**
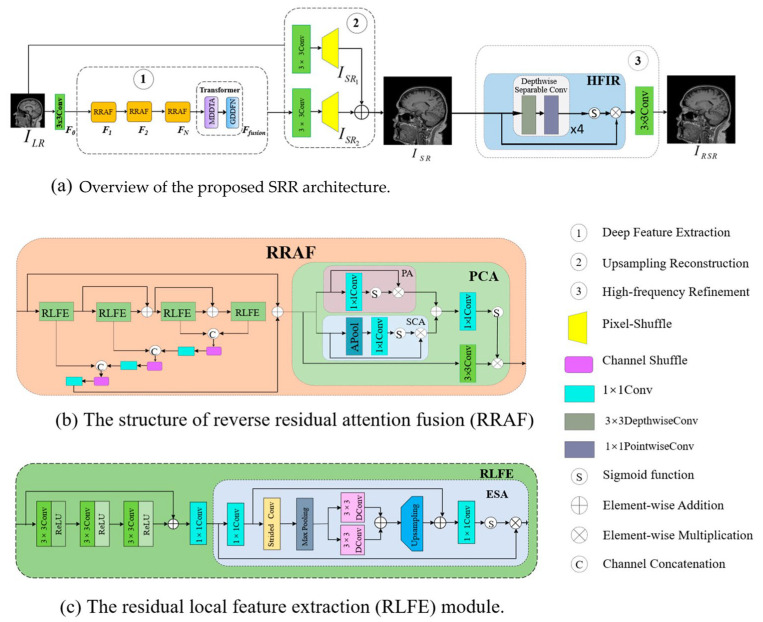
Overview of the proposed CHARMS framework, consisting of the following three key components: deep feature extraction, upsampling reconstruction, and high-frequency refinement (**a**). Deep feature extraction involves a stack of Reverse Residual Attention Fusion (RRAF) blocks (**b**), each incorporating four improved residual local feature extraction (RLFE) modules (**c**) with Enhanced Spatial Attention (ESA). A Multi-Depthwise Dilated Transformer Attention (MDDTA) transformer with supporting GDDFN further enhanced features. Various attention mechanisms, including ESA, pixel attention (PA), and shallow channel attention (SCA), are integrated into architecture. Solid arrows indicate data/model flow.

The proposed CHARMS network reconstructs HR MR images from LR inputs through an end-to-end mapping

(1) $$I_{SR}=f_{\theta }\left(I_{LR}\right),$$

where $f_{\theta }$ denotes the trainable parameters of the model. As illustrated in Figure 1, CHARMS comprises three sequential stages as follows: (1) RRAF blocks [11] for local texture encoding and multi-scale spatial awareness; (2) contextual enhancement through a MDDTA layer [14] coupled with a Gated Depthwise Dilated Feed-Forward Network (GDDFN) [52] to capture long-range dependencies; and (3) reconstruction and refinement that combines pixel-shuffle upsampling [53] with a High-Frequency Information Refinement (HFIR) module [54] to restore fine structural details. A global residual connection adds shallow features directly to the final output, preserving low-frequency signal fidelity and improving optimization stability. Each design element within CHARMS was introduced to address the performance–efficiency trade-off that limits most existing CNN-Transformer hybrid SR models.

Unlike deep CNNs [35,36,37,38,39,55] that rely on large parameter counts or pure Transformers [30,31,32,33] that suffer from high computational complexity, CHARMS unifies convolutional locality and Transformer globality in a lightweight yet expressive architecture. Its design incorporates the following three principal innovations relative to existing lightweight SR architectures: (1) RRAF modules were adapted and extended from the reverse-attention principle introduced in prior work [11], are reformulated for MRI SR by integrating residual feature refinement, channel shuffle, and enhanced spatial attention to improve feature reuse and gradient flow while directing focus toward anatomically salient regions; (2) an attention regularization mechanism, applied jointly across the CNN and Transformer branches, suppresses redundant activations and stabilizes optimization, reducing overfitting and improving generalization across contrasts and scanners; (3) the MDDTA layer [14] introduces depthwise-separable projections and dilated attention heads to maintain linear computational complexity while preserving long-range contextual modeling, which is essential for capturing extended anatomical structures.

Through these interdependent innovations, CHARMS achieves a balance between representational richness and computational efficiency, reducing model size and inference time while improving fidelity and structural sharpness. The following subsections describe each architectural component, dataset preparation, and experimental protocols used for model training and evaluation.

### 3.2. Reverse Residual Attention Fusion (RRAF) Block

At the core of CHARMS lies the RRAF module [11], which integrates residual learning and spatial attention to extract discriminative features while maintaining parameter efficiency. As shown in Figure 1b, each RRAF block contains several RLFE units [56] composed of paired convolutions with ReLU activations and an ESA operator. ESA generates a spatial weight map that highlights high-frequency anatomical regions such as tissue interfaces and structural boundaries. Dilated convolutions with rates of 1 and 2 expand the receptive field, while a channel-reduction and recovery sequence captures multi-scale dependencies. To enhance inter-channel communication without increasing parameters, channel shuffle operations are incorporated, allowing independent feature groups to exchange information efficiently. Mathematically, the output of an RRAF block can be expressed as:

(2) $$F_{out}=\mathrm{Shuffle}(\sum_{k=1}^{K}RLFE_{k}(F_{in})+F_{in}),$$

where $F_{in}$ and $F_{out}$ represent the input and output feature maps, *k* is the number of RLFE units, and Shuffle(⋅) denotes channel shuffling. Equation (2) provides an overview, with details in (3) for RLFE summation and (5) for channel shuffle to enhance inter-feature communication. This design facilitates both spatial adaptivity and gradient stability, addressing the common vanishing gradient issues observed in deeper CNNs. Each RRAF block aggregates K RLFE modules [56] that jointly encode spatial detail and contextual cues. For the i-th block,

(3) $$F_{\mathrm{RRAF}}=F_{in}+\sum_{k=1}^{K}\varepsilon_{k}(F_{k-1}),F_{0}=F_{in},$$

where $\varepsilon_{k}(\cdot )$ denotes an RLFE unit consisting of two 3×3 convolutions with ReLU activation followed by an ESA operation. ESA computes a spatial weight map

(4) $$A_{s}=\sigma \;(C_{↑}(C_{d2}(C_{d1}(P(C_{↓}(F)))))),$$

with $C_{↓}$ and $C_{↑}$ representing channel-reduction and recovery layers, $C_{d1}$ and $C_{d2}$ dilated convolutions of rates 1 and 2, and σ the sigmoid activation. The refined output $F^{\prime }=F⊙A_{s}$ selectively enhances informative regions, while a channel-shuffle operator S(⋅) intermixes channel groups to strengthen cross-feature communication without increasing parameter count:

(5) $$F_{\mathrm{RRAF}}\leftarrow S\left(F_{\mathrm{RRAF}}\right).$$

Residual skip connections maintain integrity and mitigate gradient attenuation.

### 3.3. Pixel–Channel Attention (PCA) Module

To further recalibrate feature saliency, CHARMS employs a PCA mechanism that merges pixel-level and channel-level cues. Given an input tensor F, pixel attention is computed as

(6) $$A_{p}=\sigma \left(C_{1\times 1}\left(F\right)\right),$$

while channel attention is

(7) $$A_{c}=\sigma (C_{1\times 1}(\mathrm{AvgPool}(F))).$$

The combined attention map

(8) $$A_{\mathrm{PCA}}=N\left(A_{p}+A_{c}\right)\;$$

is normalized and applied elementwise, yielding the recalibrated feature

(9) $$F_{\mathrm{PCA}}=F⊙A_{\mathrm{PCA}}.$$

This joint weighting emphasizes anatomically relevant structures and suppresses background noise with negligible computational cost.

### 3.4. Transformer Module with MDDTA and GDDFN

Local convolutions cannot effectively capture long-range anatomical dependencies; thus, CHARMS introduces a lightweight Transformer composed of a Multi-Depthwise Dilated Transformer Attention (MDDTA) layer [14] followed by a Gated Depthwise Dilated Feed-Forward Network (GDDFN) [52]. The intermediate feature map obtained after the MDDTA block (plus its residual skip connection) is

(10) $$F_{t}=A_{\mathrm{MDDTA}}\left(F_{in}\right)+F_{in},$$

The combined process after GDDFN block can be expressed as

(11) $$F_{out}=G_{\mathrm{GDDFN}}\left(F_{t}\right)+F_{t}$$

The MDDTA computes self-attention over multi-scale depthwise features:

(12) $$A_{\mathrm{MDDTA}}(F_{in})=\sum_{i}w_{i}\;\mathrm{Softmax}\;(\frac{Q_{i}K_{i}^{⊤}}{\sqrt{d}})V_{i},Q_{i},K_{i},V_{i}=C_{r_{i}}(F_{in}),$$

where $C_{r_{i}}$ is a depthwise convolution with dilation $r_{i}$ and $w_{i}$ are learned weights. This formulation expands the receptive field while maintaining linear complexity. The subsequent GDDFN enhances nonlinearity through gated depthwise convolutions:

(13) $$G_{\mathrm{GDDFN}}(F)=C_{1\times 1}\;[\phi (C_{d}(F_{1}))⊙C_{d}(F_{2})],$$

where ϕ denotes GELU activation. Together, these components effectively balance contextual modeling power and efficiency.

### 3.5. High-Frequency Information Refinement (HFIR)

After pixel-shuffle upsampling [53], the HFIR module [54] performs residual correction to restore attenuated high-frequency information. The final reconstruction can be written as

(14) $$I_{SR}=U\left(F_{out}\right)+\sigma \left(C_{3\times 3}^{\left(4\right)}\left(U\left(F_{out}\right)\right)\right)⊙U\left(F_{out}\right),$$

where U denotes the upsampling operator, $C_{3\times 3}^{\left(4\right)}$ represents four stacked depthwise separable convolutions, and σ is a sigmoid gate. This refinement selectively boosts edge and texture components while suppressing reconstruction artifacts.

### 3.6. Datasets and Preprocessing

To evaluate the performance and generalization capability of CHARMS framework, we employed four publicly available brain MRI datasets that encompass diverse imaging contrasts, spatial resolution, and acquisition protocols. As summarized in Table 1, the datasets used in this study are from the Human Connectome Project Young Adult study (HCP-YA) [16], IXI dataset [15], and two sets (PTT1 and PTT2) of paired 3T/7T studies [17,18]. The HCP-YA dataset offers high-quality 3T scans at 0.7 mm isotropic resolution for a relatively homogeneous healthy control (HC) cohort, the IXI dataset provides T1W and T2W volumes (0.94 × 0.94 × 1.2 mm^3^) of more heterogenous participants over different platforms (1.5T to 3T of different vendors), and the two paired 3T/7T datasets supply matched cross-field T1W and T2W images, where the 7 T data serve as pseudo-ground-truth for cross-field validation. To train the CHARMS model, all 3D image volumes were bias-field corrected [57] with N4ITK and skull-stripped using SynthStrip [58]. This bias-field correction was applied consistently to all volumes across the IXI, HCP-YA, PTT1, and PTT2 datasets to ensure uniform preprocessing. The central 100 slices were downsampled by factors ×2 and ×4 using bicubic interpolation to form LR–HR pairs. Datasets were split according to the ratio of 70:20:10 for training, validation, and testing at the subject level, with intensities normalized to [0, 1]. Specifically, intensity normalization was performed via min–max scaling applied independently to each volume (i.e., rescaling voxel intensities based on the minimum and maximum values within that volume), without additional techniques such as Z-score normalization, White Stripe, or histogram matching between datasets. This approach was used consistently across all datasets to maintain reproducibility and support model generalization, as no direct quantitative comparisons were made between datasets.

### 3.7. Training Protocol and Comparative Models

All experiments were implemented in PyTorch 2.0 and executed on an NVIDIA RTX 4090 GPU (24 GB GDDR6X VRAM) under standard conditions (PyTorch 2.0, CUDA 11.7, and no additional optimizations such as TensorRT). Model optimization employed the AdamW optimizer [59] with $\beta_{1}=0.9$, $\beta_{2}=0.999$, and $\epsilon =10^{-8}$ hyper parameters suggested in the literature [60,61,62]. Training minimized an $L_{1}$ reconstruction loss over 200 epochs [63] with a batch size of 16 and an initial learning rate of $10^{-4}$. The proposed CHARMS network comprises approximately 1.9 million parameters and requires about 30 GFLOPs for a 256 × 256 input, yielding roughly 40% faster inference than the widely used EDSR model [6] while achieving higher reconstruction fidelity across all benchmark datasets.

To ensure a fair and reproducible evaluation, CHARMS was compared with the following seven representative 2D SR networks that together span the evolution of deep SR approaches: the non-learned bicubic interpolation baseline; early CNN architectures SRCNN [4], VDSR [5], and EDSR [6]; the attention-augmented CNN PAN [12]; and the more recent multi-scale and attention-driven hybrids W2AMSN-S [64] and FMEN [65]. All baseline models were trained from scratch on identical training, validation, and testing partitions using the same optimizer, learning-rate schedule, and number of epochs, ensuring consistent experimental conditions across architectures.

Performance was assessed using the Peak Signal-to-Noise Ratio (PSNR) [66] and Structural Similarity Index (SSIM) [67] to quantify both numerical accuracy and perceptual quality. In addition, qualitative visual analysis was conducted to evaluate the recovery of fine anatomical structures, such as cortical boundaries and tissue interfaces. Computational efficiency was measured in terms of inference time and parameter count using standardized 256 × 256 MR image inputs. Finally, a comprehensive ablation study isolated the contributions of the RRAF, PCA, MDDTA, and HFIR modules, demonstrating how each component contributes to CHARMS overall balance between efficiency and reconstruction quality.

### 3.8. Cross-Field Adaptation and Evaluation Procedure

**Figure 2:**
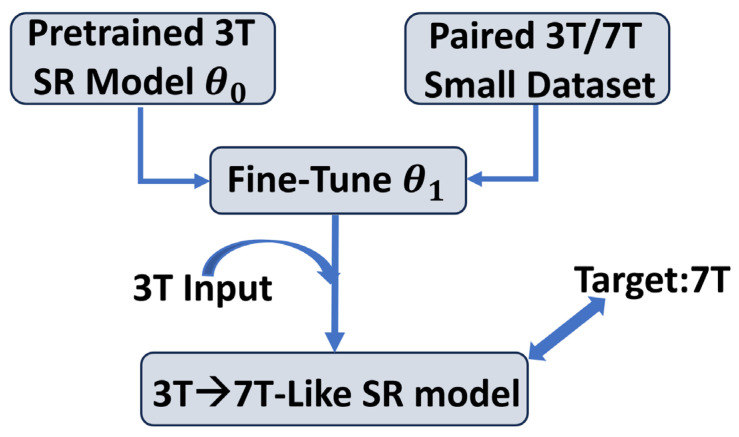
Schematic of the CHARMS cross-field adaptation workflow. Solid arrows indicate data/model flow; The curved blue arrow represents the fine-tuning process using paired 3T/7T data; the final inference path (3T input → 7T-like output) is shown with a thick arrow.

To adapt CHARMS for generating 7T-like images from 3T inputs, we performed cross-field fine-tuning using the PTT1 dataset [17] of 20 subjects (see Table 1), each providing matched 3T/7T T1-weighted and T2-weighted volumes. The overall procedure is illustrated in Figure 2.

The pretrained model $f_{\theta_{0}}$ is fine-tuned on 20 paired 3T/7T subjects from PTT1 dataset to obtain $f_{\theta_{1}}$, which is then evaluated on the PTT2 dataset [17] of 10 subjects to assess cross-site generalization. Let

(15) $$D_{\mathrm{ft}}={\left\{[(x_{i}^{3T},\;y_{i}^{7T})]\right\}}_{i=1}^{20}$$

denote this fine-tuning dataset and let $f_{\theta_{0}}$ represent the pretrained CHARMS model obtained from large-scale multi-contrast MRI SR training described above. Fine-tuning adapts the model parameters to produce an updated model $f_{\theta_{1}}$ by solving

(16) $$\theta_{1}=arg\;\underset{\theta }{min}\sum_{(x_{i}^{3T},y_{i}^{7T})\in D_{\mathrm{ft}}}L(f_{\theta }(x_{i}^{3T}),\;y_{i}^{7T}),$$

where the total training loss for cross-field adaptation was defined as

(17) $$L=\lambda_{1}∥f_{\theta }\left(x_{i}^{3T}\right)-y_{i}^{7T}{∥}_{1}+\lambda_{2}\left(1-SSIM\left(f_{\theta }\left(x_{i}^{3T}\right),y_{i}^{7T}\right)\right)+\lambda_{3}L_{\mathrm{AR}}$$

includes the pixel-wise $L_{1}$ reconstruction term, a structural-similarity term, and the proposed attention-regularization (AR) penalty to enhance fine-scale anatomical fidelity. To mitigate overfitting on the 20-subject fine-tuning dataset. We used fixed weights $\lambda_{1}=1$, $\lambda_{2}=0.1$, and $\lambda_{3}=0.005$. We fine-tuned the model using a reduced learning rate of $10^{-5}$ and restricted trainable parameters to the last two Transformer blocks and the decoder, while keeping the early CNN backbone frozen. This selective fine-tuning strategy follows established transfer-learning principles, where early layers capture stable, generalizable features while later layers adapt to domain-specific variations [68]. In medical imaging, partial fine-tuning of deeper layers has been shown to be more effective and less prone to overfitting than full retraining when data are limited [69].

Model evaluation was conducted using the independent external dataset PTT2 [18] consisting of 10 additional subjects with paired 3T/7T volumes.

(18) $$D_{\mathrm{test}}=[(x_{j}^{3T},\;y_{j}^{7T}){]}_{j=1}^{10}.$$

This strict separation of sites ensures that the evaluation reflects cross-site generalization rather than memorization of vendor- or protocol-specific features. For each subject in the external cohort, from the 3T inputs, the adapted model produced a 7T-like prediction

(19) $${\hat{y}}_{j}^{7T}=f_{\theta_{1}}\left(x_{j}^{3T}\right),$$

which was quantitatively compared against the true 7T reference $y_{j}^{7T}$ using PSNR, SSIM, and additional region-specific sharpness and edge-contrast metrics. Qualitative assessment emphasized cortical ribbon delineation, deep-gray-matter contrast, and cerebellar structural clarity.

## 4. Results

### 4.1. Benchmark Performance

**Figure 3:**
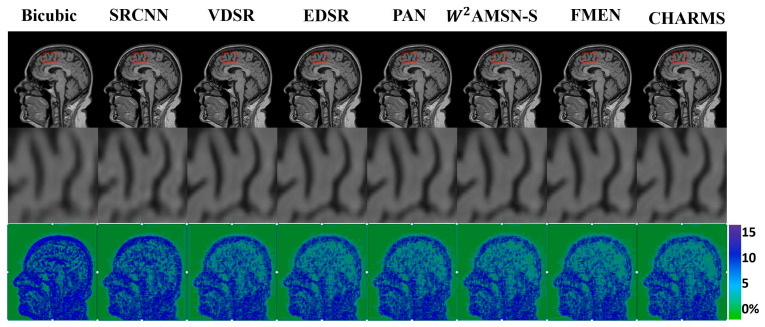
Qualitative ×4 super-resolution results on a representative sagittal IXI T1w slice. **Top** row: full-slice reconstructions showing overall image quality and contrast, with red rectangles indicating zoomed regions. **Middle** row: zoomed regions highlighting fine anatomical details. **Bottom** row: MSE maps with coluber visualizing residual errors and potential artifacts.

The proposed CHARMS network was first evaluated against seven representative SR methods—Bicubic, SRCNN [4], VDSR [5], EDSR [6], PAN [12], W2AMSN-S [64], and FMEN [65]—on the IXI (T1w/T2w) and HCP-YA (T1w) datasets. Figure 3 presents qualitative comparisons for a representative sagittal slice of the IXI T1w volume downsampled by a factor of four. CHARMS visibly restores sharper cortical and subcortical structures with reduced blurring, while competing models exhibit varying degrees of texture loss or over-smoothing.

Quantitative results for ×2 and ×4 up-scaling are summarized in Table 2 and Table 3. At ×2 scaling, CHARMS achieves PSNR/SSIM values of 37.79 dB/0.973 on IXI T1w and 38.56 dB/0.981 on IXI T2w, outperforming FMEN and W2AMSN-S by ≈0.2–0.6 dB with fewer parameters. At ×4 scaling, CHARMS yields 33.27 dB/0.945 on IXI T1w and 32.97 dB/0.956 on IXI T2w, again slightly higher than the best competing networks. On the high-quality HCP dataset, performance differences are smaller but consistent: CHARMS matches or exceeds FMEN while maintaining the lowest model complexity (≈1.9 M parameters).

These results confirm that CHARMS attains a favorable trade-off between reconstruction fidelity and computational efficiency. Its complementary convolutional and Transformer branches allow the network to preserve fine anatomical textures while modeling extended contextual relationships. By contrast, purely convolutional baselines (SRCNN, VDSR, EDSR, and PAN) either fail to recover global continuity or require deeper architectures with higher latency.

**Figure 4:**
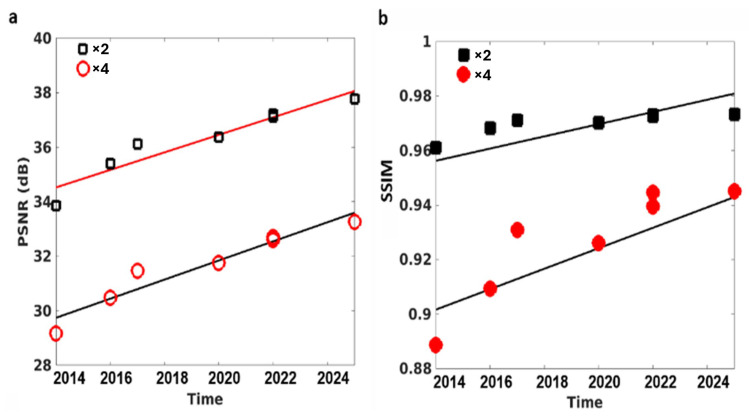
Evolution of super-resolution performance on the IXI T1w dataset from 2014 to 2025 [35,43,44,45,46,47,51,70,71,72,73,74,75,76]. (**a**) Peak Signal-to-Noise Ratio (PSNR, dB) and (**b**) Structural Similarity Index (SSIM) for ×2 (black) and ×4 (red) upscaling factors. Solid lines represent linear regression fits to published state-of-the-art results compiled from the literature over this period, illustrating the steady progress in reconstruction fidelity. Individual data points (filled squares for ×2, filled circles for ×4) correspond to the validated performance of models evaluated in the current study, including CHARMS. CHARMS achieves state-of-the-art fidelity for both scaling factors while maintaining substantially lower computational complexity.

The IXI T1w dataset has served as a standard benchmark for MRI super-resolution since 2014, enabling direct longitudinal comparison of model performance across studies [35,43,44,45,46,47,51,70,71,72,73,74,75,76]. Figure 4 summarizes the evolution of reconstruction fidelity over this period, plotting reported PSNR (Figure 4a) and SSIM (Figure 4b) values for ×2 and ×4 upscaling from the literature, alongside linear regression trends. Both metrics exhibit a consistent, approximately linear improvement over time, reflecting ongoing advances in deep learning architectures and training strategies. The models evaluated in the current study, including CHARMS, closely align with these established trends. Notably, CHARMS reaches state-of-the-art fidelity levels for both scaling factors—matching or exceeding recent heavyweight baselines—while offering superior computational efficiency (~1.9 M parameters and ~30 GFLOPs versus substantially higher values for competing SOTA methods). This demonstrates that CHARMS achieves cutting-edge performance without relying on increased model complexity, highlighting the effectiveness of its lightweight hybrid design and attention regularization.

To assess the contribution of each component, an ablation study was conducted on the IXI T1w and T2w datasets at ×2 and ×4 scaling. The baseline model consisted of stacked RRAF blocks without channel shuffle, PCA, Transformer, or HFIR modules. Successive additions of these components (Table 4) show a progressive improvement in both PSNR and SSIM. Introducing channel shuffle alone increased PSNR by about 1 dB, highlighting its effectiveness in promoting inter-channel interaction. Adding the PCA module yielded an additional 0.1 dB gain and improved structural similarity, confirming the advantage of combined pixel- and channel-level calibration. The inclusion of the Transformer further enhanced performance by ≈0.02–0.03 dB, reflecting improved long-range feature modeling. The full CHARMS model, integrating all modules including HFIR, achieved the highest metrics across all datasets and scaling factors with only marginal increases in parameter counts (<2 MB).

### 4.2. Ablation Study

**Figure 5:**
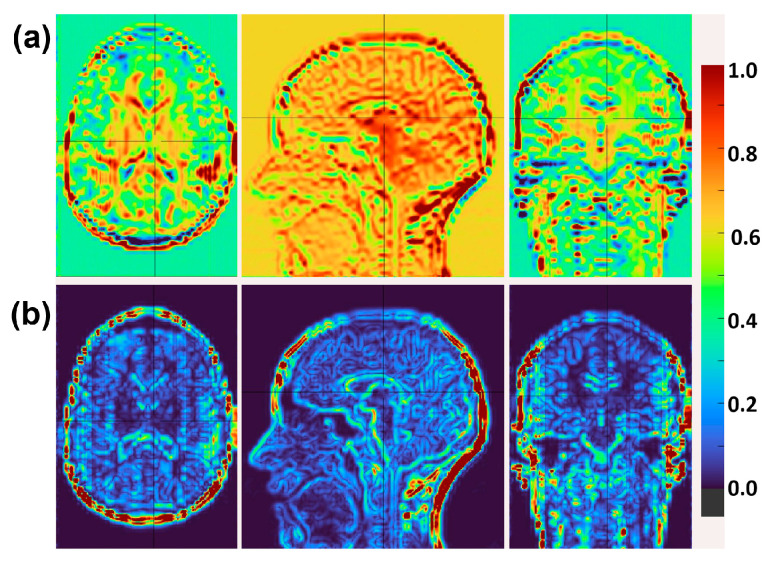
Visualization of attention maps from the Multi-Depthwise Dilated Transformer Attention (MDDTA) block on representative axial slices from the IXI T1w dataset. (**a**) Attention maps before attention regularization, showing relatively diffuse and less structured activation patterns. (**b**) Attention maps after regularization, demonstrating sharper and more focused attention concentrated along anatomically salient regions, such as cortical boundaries, sulci, and white-gray matter interfaces. Crossing black lines indicate the positions of the orthogonal cross-sections (axial, sagittal, and coronal views) displayed in each column.

To further illustrate the effect of the proposed attention regularization, Figure 5 shows attention maps extracted from the MDDTA block before and after applying the regularization loss on representative IXI T1w slices. Without regularization (Figure 5a), attention is distributed more diffusely across the entire image, with limited selectivity toward structural boundaries. In contrast, after regularization (Figure 5b), attention becomes markedly more focused and structured, preferentially highlighting anatomically salient regions including cortical folds, sulci, and white-gray matter junctions. This sharper localization demonstrates that the regularization successfully suppresses redundant activations and encourages the model to efficiently direct its focus to informative features critical for high-fidelity super-resolution, contributing to the observed improvements in both quantitative metrics and perceptual quality.

Although AR yields the smallest isolated PSNR improvement (~0.05 dB), it plays a crucial role in suppressing redundant attention patterns and stabilizing optimization. This is especially valuable in the resource-limited cross-field fine-tuning scenario, where AR contributes to consistent high-fidelity 7T-like reconstructions without overfitting on the small twenty-subject dataset.

**Figure 6:**
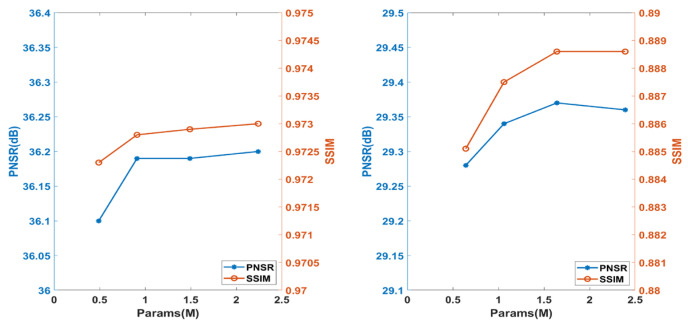
Performance on the IXI dataset (T1w) across different model configurations.

Figure 6 illustrates the influence of network depth, parameterized by the number of RRAF and RLFE blocks. Performance saturated beyond a (4, 4) configuration, while deeper variants provided negligible gains at substantially higher computational cost. This observation guided the final model design used throughout subsequent experiments.

Consistent with backbone depth saturation, additional experiments evaluated stacking multiple MDDTA blocks or varying their position. Stacking 2–3 blocks provided marginal PSNR gains (<0.01 dB) at ~15–30% higher computational cost, while earlier placement was less effective due to insufficient local feature maturity. The selected single late-stage MDDTA thus achieves the optimal balance for lightweight clinical MRI SR.

### 4.3. Cross-Field Validation Using Paired 3T/7T Datasets

To verify generalization beyond synthetic downsampling, CHARMS was further tested on two datasets of paired 3T/7T (PTT) MR images [17,18]. Models pre-trained on IXI and HCP-YA datasets were fine-tuned using the aligned 3T inputs and 7T ground-truth targets. CHARMS successfully synthesized 7T-like images from clinical 3T acquisitions (Figure 5), achieving remarkable quantitative gains over the original 3T images as follows: an average PSNR improvement of ≈6 dB across T1w and T2w volumes (Table 5) and an SSIM increase of 0.12 (Table 6)—a particularly large leap.

Qualitatively, Figure 5 presents representative axial T1w slices before and after super-resolution. The super-resolved images display markedly sharper cortical gyri, crisper gray-white matter boundaries, and enhanced tissue contrast, closely approximating the visual appearance of true 7T acquisitions. Quantitative consistency is equally striking. Swarm plots in Figure 6 and the summary statistics in Table 6 reveal that the per-slice SSIM distribution for T1w data becomes dramatically narrower after super-resolution, with standard deviation reduced by >50%. This collapse of variance indicates highly reliable structure and texture recovery across diverse anatomical regions.

These results demonstrate that CHARMS effectively bridges the resolution gap between clinical 3T and ultra-high field 7T MRI without requiring paired 7T data during deployment. The ability to upgrade standard-of-care 3T scans to near-7T quality highlights the framework’s strong generalization across magnetic field strengths, scanner vendors, and acquisition protocols—paving the way for its application to low-field and portable MRI systems, where hardware-imposed resolution limitations remain a major challenge.

**Figure 7:**
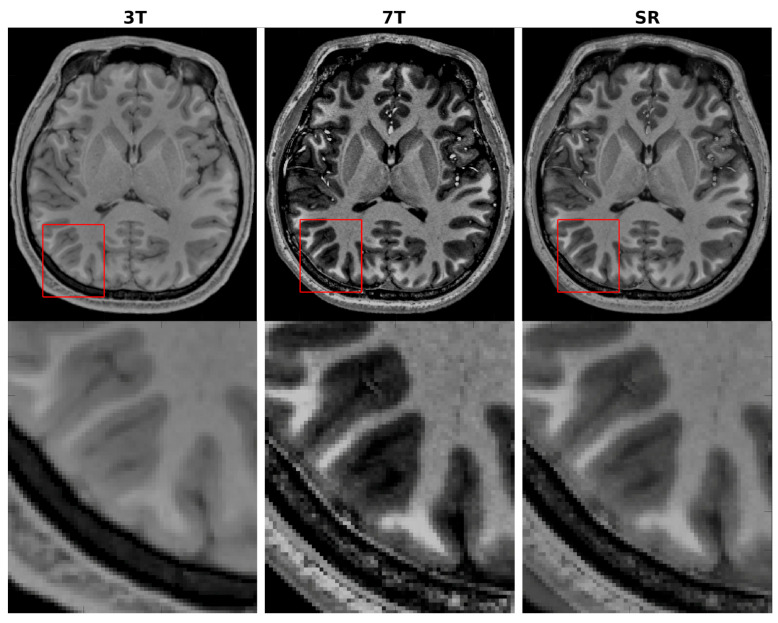
A representative T1w axial slice of the brain as well as a 4-fold zoomed-in display of the selected region marked with a red box showcasing the application of the proposed CHARMS network for SR reconstruction on the 3T MRI data using the paired PTT dataset (PTT2).

**Figure 8:**
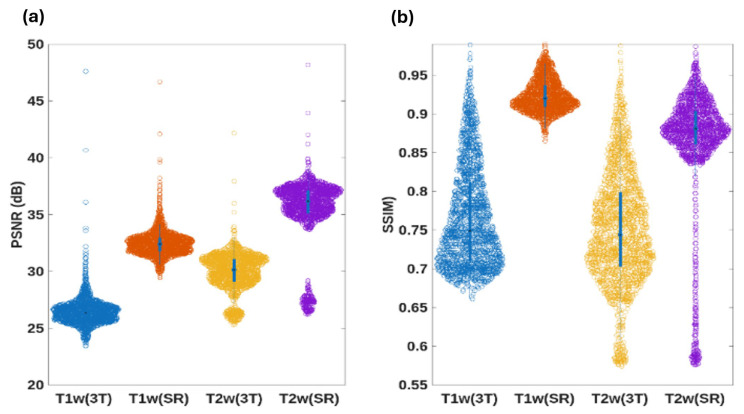
Swarm boxplot of PSNR (**a**) and SSIM (**b**) metrics for the T1w, T2w, and their corresponding SR based on the fine-tuned SR framework using the PTT datasets. Colors were used to distinguish the different datasets.

To further quantify the enhancement in image quality, we evaluated Signal-to-Noise Ratio (SNR) and gray-white matter Contrast-to-Noise Ratio (CNR) on the PTT2 test set. As shown in Table 7, CHARMS SR images improve mean SNR over native 3T by ~0.8 for T1w (8.994 vs. 8.196) and ~0.9 for T2w (5.697 vs. 4.802), approaching native 7T levels (9.393 for T1w, 6.198 for T2w). Similarly, Table 8 reveals CNR gains of ~0.1 for T1w (2.209 vs. 2.103) and ~0.7 for T2w (2.631 vs. 1.901), closely matching 7T values (2.297 for T1w, 2.825 for T2w). These metrics objectively confirm the SR outputs’ ‘near-7T quality’ in terms of reduced noise and improved tissue differentiation, see Figure 7 and Figure 8.

In addition to reconstruction accuracy, CHARMS delivers substantial computational advantages. With ≈1.9 million parameters and ≈30 GFLOPs per 256 × 256 input, it is significantly lighter than W2AMSN-S (11 M) and FMEN (3.8 M) while producing higher PSNR and SSIM. Training time was ≈7 h per configuration, compared with 14 h for W2AMSN-S. Inference on a single RTX 4090 required ≈11 ms per slice. For a typical 3D brain volume (e.g., 176 slices in PTT datasets), accounting for slices without brain tissue via foreground masking, total inference time is approximately 1.6–1.9 s on an RTX 4090 GPU—enabling near-real-time processing in clinical reconstruction pipelines.

The observed efficiency stems from the combined use of depthwise separable convolutions, channel shuffle, and attention regularization. Depthwise operations reduce parameter count and memory usage, while the attention regularization strategy limits redundancy in learned attention maps, improving convergence stability and generalization. The resulting architecture thus achieves the accuracy of more complex Transformer networks with the speed of lightweight CNNs—an essential property for integration into resource-constrained imaging systems or embedded sensor pipelines.

## 5. Discussion

The proposed CHARMS framework demonstrates that a carefully engineered CNN-Transformer hybrid can achieve an effective balance between reconstruction accuracy, computational efficiency, and architectural interpretability in MRI SR reconstruction. By pairing convolutional local feature extraction with Transformer-based global context modeling, CHARMS consistently outperformed both classical CNN models and pure Transformer architectures across multiple datasets and scaling factors. The combination of RRAF [11] and MDDTA [14] proved particularly powerful in preserving fine-grained textures while maintaining global anatomical coherence in the reconstructed images.

A central contributor to the performance of CHARMS is its attention regularization mechanism [7,23,37,52,70,77,78], which suppresses redundant activation patterns and stabilizes optimization. Existing hybrid SR models such as SwinIR [28] and Restormer [27] often rely on densely parameterized attention blocks that encode overlapping contextual information, resulting in diminishing returns when scaling to high-resolution MRI. In contrast, CHARMS encourages diverse and complementary attention maps, enabling sharper delineation of tissue interfaces, improved structural consistency, and reduced over-smoothing—limitations frequently observed in CNN-based SR frameworks such as EDSR [6] and PAN [12]. By referencing 2023–2025 studies [35,42,43,44,45,46,47,51], CHARMS’ novel attention regularization and MDDTA block fill gaps in stable, low-data adaptation, enabling near-7T quality from 3T inputs with minimal overhead.

The marginal direct fidelity gain from AR in standard training is outweighed by its stabilization effects and negligible computational cost, making it a worthwhile addition for reliable performance in diverse clinical settings, including low-data adaptation regimes.

The hybrid nature of CHARMS provides a principled solution over simpler pure architectures as follows: ablation results (Table 4) show that removing the Transformer component degrades PSNR/SSIM by ~0.02–0.06 dB, underscoring its role in enhancing global coherence without the overhead of full Transformer models. In brain MRI, where scans exhibit sparse, hierarchical patterns, this combination yields sharper, more anatomically consistent reconstructions than optimized pure CNNs (e.g., outperforming EDSR by ~0.3 dB PSNR) or Transformers, prioritizing efficiency for practical deployment.

Although diffusion-based models [41,42,43] and heavily parameterized vision/state-space-model hybrids [40] have shown excellent perceptual quality in recent studies, they typically require iterative sampling (10–100× slower) or >10–50 million parameters. Because the primary goal of CHARMS is lightweight single-pass inference suitable for clinical and resource-constrained environments, direct head-to-head comparison with these fundamentally different paradigms was outside the scope of this study.

Addressing RQ1, CHARMS’ hybrid architecture (Section 3.1, Section 3.2, Section 3.3, Section 3.4 and Section 3.5) outperforms baselines like EDSR and FMEN by 0.1–0.6 dB PSNR with ~40% faster inference (Table 2 and Table 3), validated on IXI and HCP-YA datasets. For RQ2, attention regularization stabilizes optimization, yielding robust gains in ablation studies (Table 4) and cross-contrast performance. RQ3 is demonstrated through cross-field adaptation (Section 4.3), achieving ~6 dB PSNR and 0.12 SSIM improvements over native 3T, with SNR/CNR nearing 7T levels (Table 5, Table 6, Table 7 and Table 8), paving the way for reduced scan times in clinical workflows.

In the cross-field adaptation results (Table 5 and Table 6), we observed greater variability in performance metrics (e.g., larger standard deviations in PSNR and SSIM) for T2w reconstructions compared to T1w results. This increased variability in T2w contrasts is likely attributable to inherent tissue contrast characteristics as follows: T2w imaging is more sensitive to fluid content, edema, and subtle tissue–water variations, which can introduce higher slice-to-slice heterogeneity in anatomical detail, noise patterns, and edge sharpness—particularly in downsampled inputs. In contrast, T1w images typically exhibit more consistent gray-white matter differentiation and lower sensitivity to such fluid-related variations, leading to more stable reconstruction outcomes. Additionally, the paired 3T/7T datasets (PTT1/PTT2) contain fewer T2w volumes relative to T1w in some splits, potentially contributing to slightly reduced robustness during fine-tuning. These observations align with prior studies noting greater challenges in modeling T2w contrasts due to their pronounced sensitivity to relaxation properties and potential artifacts. Future multi-contrast training strategies could further mitigate this by incorporating explicit T2w-specific regularization or additional data augmentation.

**Figure 9:**
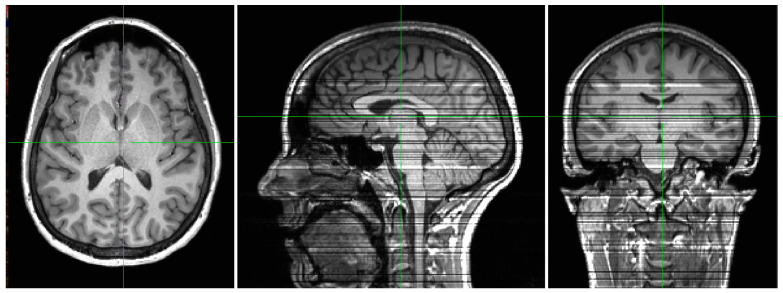
Qualitative illustration of the limitation of the 2D slice-wise processing in CHARMS. The (**left**) panel shows an axial view of a representative super-resolved T1w volume from the IXI dataset. The (**middle** and **right**) panels display reconstructed sagittal and coronal views, respectively, obtained by resampling the 3D volume along the orthogonal planes indicated by the green crosshairs. Horizontal banding artifacts and subtle slice-to-slice intensity inconsistencies are visible in the reformatted views (most evident in the sagittal and coronal planes), highlighting the lack of explicit through-plane continuity modeling in the current 2D approach.

Despite its strengths, CHARMS currently operates in a 2D slice-wise manner. Extending it to native 3D processing [70,79] would better exploit through-plane continuity and further improve consistency. While CHARMS achieves state-of-the-art performance with a lightweight 2D slice-wise architecture, this design inherently neglects explicit modeling of through-plane continuity. As a result, minor inconsistencies can arise across slices in the reconstructed 3D volume. Figure 9 illustrates this limitation on a representative ×4 super-resolved IXI T1w scan. Although the axial view (native processing plane) appears smooth and artifact-free, reformatted sagittal and coronal views reveal subtle horizontal banding and slight intensity variations between adjacent slices (visible as faint striped patterns). These discontinuities, while minor and not clinically disruptive in most cases, could be further mitigated by extending the model to full 3D processing or incorporating inter-slice consistency constraints in future work.

Additionally, while supervised training with paired data yielded excellent results, incorporating self-supervised [80,81,82,83,84,85,86,87] or physics-informed strategies [43,88,89,90,91,92,93] could reduce dependency on high-quality ground-truth pairs and enhance robustness across scanners and pathologies.

While VGG-based perceptual losses are common in natural image super-resolution, we deliberately omitted them to prioritize quantitative fidelity and anatomical accuracy in clinical MRI reconstructions, relying instead on L1 and structural similarity terms to achieve high PSNR/SSIM and artifact-free results.

Although primary benchmarks employ isotropic scaling, the cross-field adaptation from clinical (often anisotropic) 3T acquisitions to near-isotropic 7T quality directly demonstrates CHARMS’ applicability to real-world anisotropic MRI, a common scenario in routine scanning protocols.

While direct comparisons to other models fine-tuned on the same limited cross-field data would further isolate transfer efficiency, the substantial gains over native 3T images—consistent with evaluation protocols in prior 3T-to-7T synthesis studies—validate CHARMS’ practical value for enhancing routine clinical acquisitions.

The added SNR and CNR analyses (Table 7 and Table 8) provide rigorous quantitative support for the ‘near-7T quality’ claim, demonstrating that CHARMS not only enhances resolution but also boosts signal characteristics critical for diagnostic confidence in clinical settings.

Clinically, the combination of high fidelity, low latency (~11 ms/slice on RTX 4090), with total inference times of ~1.6–1.9 s per 3D volume on a standard high-end GPU, CHARMS supports integration into online reconstruction workflows, and small memory footprint positions CHARMS as an ideal candidate for integration into online reconstruction pipelines, low-field/portable systems [94,95,96,97,98], or accelerated protocols—ultimately helping reduce scan time, minimize motion artifacts, and improve diagnostic confidence without additional hardware. While CHARMS demonstrates strong performance on healthy and near-healthy brain scans across T1w/T2w contrasts, its generalization to brains with gross pathologies (e.g., large tumors or significant focal lesions causing substantial anatomical distortion) remains to be fully validated, as such cases may introduce domain shifts that challenge learned feature representations; future work will explore fine-tuning or domain-adaptation strategies on pathological datasets to enhance robustness in clinical neuro-oncology applications.

In summary, CHARMS represents a significant step toward practical, clinically viable deep learning SR for MRI by delivering top-tier lightweight performance today while providing a modular foundation for future 3D and unsupervised extensions. Deploying CHARMS in real-world clinical settings is feasible due to its lightweight design (~11 ms/slice, ~1.6 s/volume on RTX 4090), enabling integration into PACS or online reconstruction via PyTorch/ONNX export. Challenges include DICOM compatibility, validation on diverse scanners/pathologies, and regulatory approval (e.g., FDA for AI tools). Required extensions are 3D volumetric processing for continuity, self-supervised fine-tuning for unlabeled data, and edge deployment on low-power GPUs for portable MRI.

While the current evaluation relies on established quantitative metrics (PSNR, SSIM, SNR, and CNR) and side-by-side qualitative visual assessment by the authors (demonstrating sharper cortical boundaries, improved gray-white contrast, and reduced blurring without obvious over-sharpening or hallucination-like artifacts), a formal blinded subjective scoring by expert radiologists (e.g., using a Likert scale for diagnostic confidence, artifact presence, and perceived resolution gain) was not performed in this study. Such an evaluation would be valuable for future clinical validation, particularly when extending CHARMS to pathological cases or real-world heterogeneous clinical scans. In the present work, the absence of gross hallucinatory artifacts in the reconstructions is supported by the high structural fidelity (SSIM gains of ~0.12) and the preservation of anatomically plausible tissue interfaces across all evaluated slices.

Recent studies in brain MRI deep learning, such as Samarasinghe et al. (2025) [99], have advanced self-supervised segmentation and edge detection on multi-modal MRI using architectures like dual-decoder 3D-UNet with SimSiam pretraining. While these approaches effectively reduce annotation dependency for tumor boundary tasks, they differ fundamentally from our focus on super-resolution reconstruction. CHARMS uniquely contributes a lightweight (~1.9 M parameters) CNN-Transformer hybrid tailored for efficient MRI SR, incorporating Reverse Residual Attention Fusion, multi-depthwise dilated Transformer blocks, and novel attention regularization to achieve superior PSNR/SSIM gains and ~40% faster inference compared to prior lightweight SR models, while enabling practical cross-field 3T-to-near-7T quality enhancement—features not addressed in segmentation-oriented works.

## 6. Conclusions

This study presented CHARMS, a CNN-Transformer hybrid framework with attention regularization for SR reconstruction of MR images. By combining convolutional feature extraction with the global contextual modeling of Transformers, CHARMS achieves superior reconstruction accuracy and visual fidelity while maintaining exceptional computational efficiency. Experimental evaluations across multiple MRI datasets confirmed its consistent performance advantage over existing CNN- and Transformer-based methods. The framework’s lightweight design and modular structure make it suitable for integration into routine MRI reconstruction pipelines, offering a practical solution for accelerating image acquisition and improving diagnostic quality. Future work will extend CHARMS to 3D volumetric SR and self-supervised domain adaptation to enhance generalization across scanners, field strength, and contrasts. Overall, CHARMS represents a promising step toward intelligent, efficient, and clinically adaptable SR reconstruction of MR images.

## Figures and Tables

**Table 1:** Summary of the MRI datasets used.

Dataset	Source	Subjects	Demographics	Contrasts	Resolution (mm^3^)
HCP-YA	[16]	1206 HC	22–36 years m/f = 507/699	T1w	0.7 × 0.7 × 0.7
				T2w	0.7 × 0.7 × 0.7
IXI	[15]	563 HC	20–86 years m/f = 250/313	T1w	0.94 × 0.94 × 1.2
				T2w	0.94 × 0.94 × 1.2
PTT1	[17]	20 HC	18–25 years m/f = 10/10	T1w	1 × 1×1, 0.7 × 0.7 × 0.7
				T2w	0.9 × 0.9 × 1.9, 0.4 × 0.4 × 1
PTT2	[18]	10 HC	25–41 years m/f = 7/3	T1w	0.8 × 0.8 × 0.8, 0.7 × 0.7 × 0.7
				T2w	0.8 × 0.8 × 0.8, 0.7 × 0.7 × 0.7

**Table 2:** Summary of the model characteristics and performances (PSNR and SSIM) after 2× downsampling and SRR enhancement. All CHARMS gains over FMEN are statistically significant (*p* < 0.05).

Model	Parameters (Million)	Train (h)	PSNR (IXIT1w)	SSIM (IXIT1w)	PSNR (IXI-T2w)	SSIM (IXI-T2w)	PSNR (HCP-T1w)	SSIM (HCP-T1w)
Bicubic	—		31.68 ± 0.55	0.935 ± 0.028	32.51 ± 0.58	0.946 ± 0.025	40.19 ± 0.48	0.9874 ± 0.020
SRCNN	0.82	0.7	33.86 ± 0.42	0.961 ± 0.018	35.33 ± 0.45	0.969 ± 0.016	43.80 ± 0.38	0.9935 ± 0.014
VDSR	0.37	3.0	35.43 ± 0.35	0.968 ± 0.015	36.74 ± 0.38	0.963 ± 0.013	44.77 ± 0.32	0.9936 ± 0.011
EDSR	1.36	4.7	36.13 ± 0.28	0.971 ± 0.012	38.07 ± 0.30	0.979 ± 0.010	46.11 ± 0.25	0.9957 ± 0.010
PAN	0.78	5.0	36.38 ± 0.27	0.970 ± 0.013	37.79 ± 0.27	0.978 ± 0.011	45.71 ± 0.21	0.9954 ± 0.008
W^2^AMSN-S	11.37	14	37.12 ± 0.22	0.972 ± 0.009	38.30 ± 0.26	0.980 ± 0.008	46.43 ± 0.20	0.9961 ± 0.007
FMEN	3.80	11	37.22 ± 0.16	0.973 ± 0.008	38.40 ± 0.17	0.981 ± 0.007	46.58 ± 0.14	0.9963 ± 0.007
CHARMS	1.74	10	37.79 ± 0.11	0.973 ± 0.008	38.56 ± 0.11	0.981 ± 0.006	46.58 ± 0.10	0.9963 ± 0.006

**Table 3:** Summary of the model characteristics and performances (PSNR and SSIM) after 4× downsampling and SRR enhancement. CHARMS shows statistically significant improvements over FMEN (*p* < 0.05) and much larger gains over heavier models like W2AMSN-S (*p* < 0.01), despite ~6× fewer parameters.

Model	Parameters (Million)	Train (h)	PSNR (IXI-T1w)	SSIM (IXI-T1w)	PSNR (IXI-T2W)	SSIM (IXI-T2W)	PSNR (HCP-T1w)	SSIM (HCP-T1w)
Bicubic	—		26.17 ± 0.58	0.786 ± 0.030	26.92 ± 0.58	0.826 ± 0.025	31.57 ± 0.48	0.924 ± 0.024
SRCNN	0.82	0.5	29.18 ± 0.50	0.888 ± 0.028	30.63 ± 0.50	0.873 ± 0.022	33.96 ± 0.39	0.948 ± 0.021
VDSR	0.37	1.5	30.49 ± 0.45	0.909 ± 0.020	32.44 ± 0.46	0.890 ± 0.020	34.60 ± 0.36	0.954 ± 0.018
EDSR	1.51	2.3	31.48 ± 0.36	0.931 ± 0.018	32.53 ± 0.38	0.948 ± 0.012	36.10 ± 0.33	0.965 ± 0.011
PAN	0.92	2.7	31.76 ± 0.28	0.926 ± 0.016	32.27 ± 0.28	0.944 ± 0.011	35.90 ± 0.28	0.964 ± 0.009
W^2^AMSN-S	11.41	9.0	32.61 ± 0.22	0.939 ± 0.016	32.76 ± 0.11	0.953 ± 0.009	36.56 ± 0.20	0.968 ± 0.008
FMEN	3.95	7.0	32.72 ± 0.20	0.944 ± 0.010	32.92 ± 0.20	0.956 ± 0.007	36.81 ± 0.18	0.969 ± 0.007
CHARMS	1.89	7.0	33.27 ± 0.14	0.945 ± 0.010	32.97 ± 0.15	0.956 ± 0.007	36.65 ± 0.11	0.969 ± 0.007

**Table 4:** Summary of ablation studies, detailing the effects of each module on the PSNR (dB) and SSIM in SRR-enhanced image quality.

Model		Baseline	+CS	+CS +PCA	+CS +PCA +Transformer	Full Model
2×	Parameters (MB)	1.46	1.49	1.69	1.72	1.74
	PSNR/SSIM (IXI-T1w)	35.12/0.967	36.19/0.973	36.27/0.973	36.28/0.973	36.29/0.973
	PSNR/SSIM (IXI-T2w)	37.38/0.973	38.43/0.979	38.50/0.981	38.51/0.981	38.56/0.981
	PSNR/SSIM (HCP-T1w)	45.63/0.989	46.44/0.995	46.56/0.996	46.56/0.996	46.58/0.996
4×	Size of Parameters (MB)	1.61	1.64	1.84	1.87	1.89
	PSNR/SSIM (IXI-T1w)	28.66/0.869	29.37/0.889	29.42/0.890	29.44/0.890	29.47/0.891
	PSNR/SSIM (IXI-T2w)	29.56/0.896	30.39/0.909	30.41/0.916	30.44/0.916	30.47/0.916
	PSNR/SSIM (HCP-T1w)	35.81/0.946	36.53/0.967	36.60/0.968	36.63/0.968	36.65/0.969

**Table 5:** PSNR for the T1w, T2w, and T1/T2 image volumes acquired at 3T and the reconstructed SRR images based on the corresponding high-resolution 7T MRI data.

Subject	3T (T1w)	SR (T1w)	3T (T2w)	SR (T2w)
1	27.310	33.330	27.359	28.475
2	28.639	34.660	32.265	38.286
3	27.927	33.947	31.938	37.958
4	26.724	32.744	30.251	36.271
5	27.174	33.195	30.546	36.566
6	27.752	33.773	31.982	38.003
7	27.717	33.737	31.956	37.976
8	27.622	33.643	30.472	36.493
9	28.394	34.415	31.208	37.229
10	27.213	33.234	31.952	37.973
Mean	27.647	33.668	30.993	36.523
Std	0.579	0.579	1.476	2.923

**Table 6:** The SSI for the T1w, T2w, and T1/T2 image volumes acquired at 3T and the generated SR images are based on the corresponding high-resolution 7T MRI data.

Subject	3T (T1w)	SR (T1w)	3T (T2w)	SR (T2w)
1	0.821	0.938	0.726	0.727
2	0.870	0.954	0.872	0.948
3	0.830	0.945	0.851	0.933
4	0.807	0.933	0.783	0.890
5	0.832	0.947	0.822	0.907
6	0.844	0.949	0.829	0.920
7	0.816	0.944	0.816	0.914
8	0.836	0.949	0.817	0.911
9	0.854	0.951	0.840	0.925
10	0.818	0.939	0.825	0.914
Mean	0.833	0.945	0.818	0.899
Std	0.019	0.007	0.040	0.062

**Table 7:** The SNR for the T1w and T2w image volumes acquired at 3T, 7T, and the reconstructed SR images based on the fine-tuned model using the paired PTT1 dataset.

Subject	3T (T1w)	7T (T1w)	SR (T1w)	3T (T2w)	3T (T2w)	SR (T2w)
1	8.007	9.013	8.557	4.301	5.983	5.481
2	8.887	10.792	10.7	5.695	7.331	6.689
3	8.478	10.368	9.628	5.353	6.501	5.956
4	7.451	7.458	7.588	4.119	5.071	4.542
5	7.995	7.576	7.963	4.133	5.497	5.105
6	8.311	10.153	9.375	5.147	6.368	5.791
7	8.159	9.771	8.879	4.961	6.285	5.779
8	8.055	9.277	8.642	4.652	6.174	5.725
9	8.616	10.729	10.301	5.466	6.888	6.522
10	8.001	8.791	8.305	4.192	5.878	5.389
Mean	8.196	9.393	8.994	4.802	6.198	5.697
Std	0.401	1.201	1.003	0.601	0.652	0.631

**Table 8:** The CNR between gray and white matter brain tissues for the T1w and T2w image volumes acquired at 3T, 7T, and the reconstructed SR images based on the fine-tuned model using the paired PTT1 dataset.

Subject	3T (T1w)	7T (T1w)	SR (T1w)	3T (T2w)	3T (T2w)	SR (T2w)
1	1.916	1.915	2.081	1.551	2.823	2.357
2	3.193	3.537	3.46	2.755	4.676	3.646
3	2.559	2.557	2.492	2.431	3.321	2.520
4	1.234	1.451	1.205	1.042	1.623	1.578
5	1.393	1.679	1.307	1.298	1.722	1.742
6	2.476	2.305	2.42	2.015	2.185	2.603
7	1.953	2.285	2.21	1.986	3.082	3.398
8	1.923	2.011	2.173	1.842	3.001	2.715
9	2.607	3.421	2.718	2.549	4.174	3.416
10	1.775	1.81	2.025	1.539	1.642	2.332
Mean	2.103	2.297	2.209	1.901	2.825	2.631
Std	0.601	0.702	0.652	0.559	1.201	1.066

## Data Availability

The study was based on public domain data which are all openly accessible. Code for our preprocessing and decoding the machine learning framework will be available on GitHub. The datasets analyzed in the study were available in the public domain.

## Appendix A. Glossary of Key Acronyms

To improve readability, this appendix provides a summary table of the main acronyms used for the architectural components in the CHARMS framework.

**CHARMS**	**Full Name**	**Brief Description**
CHARMS	CNN-Transformer Hybrid with Attention Regularization for MRI Super-Resolution	The proposed lightweight model for MRI super-resolution, combining CNN and Transformer elements with attention regularization.
RRAF	Reverse Residual Attention Fusion	Backbone module for hierarchical local feature extraction, integrating residual learning and attention.
RLFE	Residual Local Feature Extraction	Units within RRAF blocks, consisting of convolutions, ReLU activations, and ESA for feature encoding.
ESA	Enhanced Spatial Attention	Spatial attention operator that highlights high-frequency regions using dilated convolutions.
PCA	Pixel–Channel Attention	Mechanism merging pixel- and channel-level attention for fine-grained feature recalibration.
MDDTA	Multi-Depthwise Dilated Transformer Attention	Transformer block for efficient long-range dependency modeling with linear complexity.
GDDFN	Gated Depthwise Dilated Feed-Forward Network	Feed-forward component in the Transformer module, enhancing nonlinearity via gated convolutions.
THFIR	High-Frequency Information Refinement	Refinement module post-upsampling to restore high-frequency details and suppress artifacts.
